# Correction: Conducting tobacco control surveys among schoolchildren in Bangladesh, India and Pakistan: A feasibility study

**DOI:** 10.1371/journal.pgph.0004602

**Published:** 2025-05-02

**Authors:** Masuma Pervin Mishu, Cath Jackson, Ann McNeill, Suneela Garg, Amod Borle, Chetana Deshmukh, M. Meghachandra Singh, Nidhi Bhatnagar, Ravi Kaushik, Rumana Huque, Fariza Fieroze, Sushama Kanan, S. M. Abdullah, Laraib Mazhar, Zohaib Akhter, Khalid Rehman, Safat Ullah, Lu Han, Anne Readshaw, Aziz Sheikh, Paramjit Gill, Kamran Siddiqi, Mona Kanaan, Romaina Iqbal

Masuma Pervin Mishu^1^, Cath Jackson^2^, Ann McNeill^3^, Suneela Garg^4^, Amod Borle^4^, Chetana Deshmukh^4^, M. Meghachandra Singh^4^, Nidhi Bhatnagar^4^, Ravi Kaushik^4^, Rumana Huque^5^, Fariza Fieroze^5^, Sushama Kanan^5^, SM Abdullah^5^, Laraib Mazhar^6^, Zohaib Akhter^6^, Khalid Rehman^7^, Safat Ullah^7^, Lu Han^2^, Anne Readshaw^2^, Aziz Sheikh^8^, Paramjit Gill^9^, Kamran Siddiqi^2^, Mona Kanaan^2^, Romaina Iqbal^6^

**1** Department of Epidemiology and Public Health, University College London, London, United Kingdom, **2** Department of Health Sciences, University of York, York, United Kingdom, **3** Addictions Department, Institute of Psychiatry, Psychology & Neuroscience (IoPPN), King’s College London, London, United Kingdom, **4** Department of Community Medicine, Maulana Azad Medical College & Associated Hospitals, New Delhi, India, **5** ARK Foundation, Dhaka, Bangladesh, **6** Department of Community Health Sciences, Aga Khan University, Karachi, Pakistan, **7** Institute of Public Health & Social Sciences, Khyber Medical University, Peshawar, Khyber Pakhtunkhwa, Pakistan, **8** Nuffield Department of Primary Care Health Sciences, University of Oxford, Oxford, United Kingdom, **9** Division of Health Sciences, Warwick Medical School. University of Warwick, Warwick, United Kingdom.
