# Supplementary material for: Conducting tobacco control surveys among schoolchildren in Bangladesh, India and Pakistan: A feasibility study
Source: PLOS Glob Public Health. 2024 Oct 3;4(10):e0003784. doi: 10.1371/journal.pgph.0003784 (PMC11449278; doi:10.1371/journal.pgph.0003784)
Supplement: S1 Text — (DOCX) [file pgph.0003784.s001.docx]

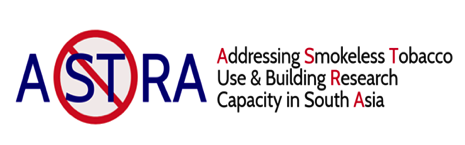


**Title of the study**: **The impact of tobacco control policies on smokeless tobacco uptake among adolescents in South Asia– longitudinal surveys.**

**Information Leaflet for Parents/Carers**

We are a team of researchers from ………………………(local institution) and the University of York, UK. We wish to conduct a school-based survey to understand the knowledge, attitude and behaviour of the adolescents towards tobacco use. We would also like to ask related questions to your son/daughter. We would also like to ask you some similar questions. We will do this survey once a year for three years.

Our aim is to study the impact of government policies on the use of smokeless tobacco among youth.

The survey is funded by the National Institute of Health Research (NIHR) in the UK. This survey has been approved by the ethics committees at …….. and ………………….(country specific research ethics committee)**,** Project ID Number:

**Name, Address and Contact Details of the Investigator**s

| Name:  Address:  Mobile no: | Name:  Address:  Mobile no: |
| --- | --- |

**We would like to invite you and your child to take part in the ASTRA study**

Before you decide whether you and your child want to take part, it is important for you to read the following information carefully and discuss it with others if you wish. Ask us/ the class teacher if there is anything that is not clear or if you would like more information.

This leaflet tells you a bit about the research to help you decide whether you would like your child to take part or not.

***I don’t know if we want our child to take part in the research.***

Your child will be asked a few simple questions about use of tobacco products. We will also ask few questions relating to parental education level, household goods and some general questions about tobacco use norm in your family.

The whole process will be completed in no more than 30 minutes. The survey will be performed by trained investigators and do not involve any risk to your child. Every possible care will be taken to cause minimum inconvenience to your child.

We will ask the same questions every year for three years.

***What will my child has to do?***

***Does my child need to do anything else?***

Your child might be invited to take part in a small meeting called as focus group discussion. The meeting will last approximately one hour. It will take place in your child’s classroom with six to eight other students. A researcher will ask him/her some questions about tobacco use and request him/her to discuss his/her thoughts and views. The meeting will be recorded on an audiotape.

There is no risk for your child to take part in this study. A disadvantage of taking part in this study is the time involved in providing responses to the questions. We will make every effort to arrange the data collection at a convenient time to minimize any disruptions to your child’s routine.

We will give your child a small incentive (school stationery) for taking part in the study.

***What are the risks and benefits to take part in the study?***

***Does my child have to take part in the ASTRA study?***

Your child does not need to take part if you don’t want him/her to. It will not change things at school at all, and you do not have to give us a reason. If you decide to take part now you can always change your mind later on. Just let us know that you do not want to take part any more.

You can withdraw your child from the study at any time even after the start. In this case, no more data will be collected. The information already collected will be kept secure and still used in the analysis unless you specifically ask for this information to be removed.

***Can I withdraw my child from the study?***

If you wish your child to take part in the study please carefully complete the parent consent form included in this information pack. Remember to write your child’s name and details on the form too. Please return the form to your child’s school within seven days after receiving this information.

***What happens next?***

We may also ask you to fill a short questionnaire. The questionnaire will be send home through your child and you would be requested to fill the questionnaire and send back to the school through your child. The question will ask about your tobacco habit and your house norm for tobacco use.

***Do I (the parents) need to take part in the study?***

If you are unsure about your child’s taking part, you can ring our research team to talk about it more or ask the class teachers. Please remember that you and your child should only participate if you want to. Your child does not have to take part if you do not want to; but we hope your child will find it interesting if he/she does. Choosing not to take part will not affect the standard of care of you or your child at school in any way.

***I’m still unsure about my child’s taking part?***

Everything that you tell us is confidential information. This means that we will not tell anyone outside the ASTRA research team what you have said. The information will not be shared with the school and would not have any impact on your child’s school record. Participation in this study is anonymous, so the name or any identifiable details will not be disclosed.

Your and your child’s name and details will be kept separate from any information that you give us. This is to avoid any other person accidently finding your details on the information that you provide.

The information you provide will be stored inside a locked cabinet. We will then securely transfer the information you provide onto a secure and password protected computer in the University of York. We will keep the information for five years and then destroy it according to the University of York policy. Audio recordings (if your child takes part in the focus group discussion) will also be handled in the strictest confidence and will only be listened to by members of the research team. The recordings will be destroyed after 5 years.

All your personal data (including audio recordings) will be handled in accordance with the General Data Protection Regulation (GDPR) and the UK’s Data Protection Act 2018. A description of GDPR can be found at the following website: <https://www.york.ac.uk/records-management/dp/>.

***How will the information and personal data me and my child give be handled?***

If you have any questions about the study please do not hesitate to contact us at the above address and telephone number. If you are unable to read and write please report us or the class teacher and we will help you to complete the form and the questionnaire. If you decide to take part you will be given this information sheet to keep and be asked to sign a consent form.

Your and your child’s participation is very important in our research and is highly valued and appreciated. Thank you for taking time to read this information leaflet and considering taking part in this research.
