# Supplementary material for: Conducting tobacco control surveys among schoolchildren in Bangladesh, India and Pakistan: A feasibility study
Source: PLOS Glob Public Health. 2024 Oct 3;4(10):e0003784. doi: 10.1371/journal.pgph.0003784 (PMC11449278; doi:10.1371/journal.pgph.0003784)
Supplement: S2 Text — (DOCX) [file pgph.0003784.s002.docx]

**Supplementary material 2 – Questionnaire**

|  |  |
| --- | --- |
| For office use only | Admin area/sub-district ID: _ _ /_ _  Union Council/Cluster ID: _ _ /_ _  Questionnaire No:  Interviewer ID No: |
|  |  |
|  |  |

| Date of completion  ..................... |  | Code of the school  .................................................................. |  | Student’s ID  ................................................ |
| --- | --- | --- | --- | --- |

**INSTRUCTIONS FOR COMPLETING THIS SURVEY**

Thank you for participating in this survey. Before you start, please read the following information, which will help you to answer the questions.

If there is a choice of answers, pick the one that is true for you and place a Tick (√) in the box next to it.

For example:

☐ Yes

🗹No

We will ask about smokeless tobacco use (tobacco that is not smoked, but is chewed or held in the mouth). We will also ask you about smoking tobacco.

**The survey has three sections:**

**Section 1: Questions about you, your home and your family**

**Section 2: Questions about smokeless tobacco**

**Section 3: Questions about smoking tobacco**

Sections 2 and 3 have further sub-sections:

a. Your tobacco use

b. Your knowledge of tobacco

c. Packaging and labelling of tobacco products

d. Awareness of tobacco

e. Advertisements or promotions for tobacco

f. Buying of tobacco products

g. Tobacco price

h. Exposure to tobacco products

**Section 2 and 3 are in two column side by side: left side is for Smokeless tobacco and right side is for smoking tobacco (cigarette, bidi etc.)**

Please complete the survey using a black/ blue ballpoint pen.

Please use your national language: Bengali/Urdu/Hindi………… number to write your ‘date of birth’ in Q1.

In other places where you need to write any number, please write the numbers in letters (eg. Five, six)

**Section 1: You, your home and your family**

1. Your date of birth

…………/………./…………

Day Month Year

☐ Don’t know

**2. How old are you?**

☐ 11 years old or younger

☐ 12 years old

☐ 13 years old

☐ 14 years old

☐ 15 years old

☐ 16 years old

☐ 17 years old or older

**3. Please indicate your sex?**

☐ Male

☐ Female

**4. In what grade/class are you?**

☐ Grade 6

☐ Grade 7

☐ Grade 8

**The next questions (Question 5 to Question 7) are about your family**

Parental education

5.1 What level of education did your mother/female carer complete?

☐ No education

☐ Primary

☐ Secondary

☐ Higher education (any education level above secondary)

5.2 What level of education did your father/male carer complete?

☐ No education

☐ Primary

☐ Secondary

☐ Higher education

6. Do your parents work to earn money?

☐ Father only

☐ Mother only

☐ Both

☐ Neither

☐ Don’t know

Does your house have the following items (Please tick the box where appropriate)

| Asset | Yes | No |
| --- | --- | --- |
| 7.1. Electricity |  |  |
| 7.2. Flush toilet |  |  |
| 7.3. Fixed telephone |  |  |
| 7.4. Cell telephone |  |  |
| 7.5. Television |  |  |
| 7.6. Radio |  |  |
| 7.7. Refrigerator |  |  |
| 7.8. Car |  |  |
| 7.9. Moped/Scooter/Motorcycle |  |  |
| 7.10. Washing machine |  |  |

**From the next part the questions are in two column side by side: left side is for Smokeless tobacco and right side is for smoking tobacco and you can give different answers for the two columns.**

**Section 2: Smokeless tobacco products**

**Section 3: Smoking tobacco products**

| **Smokeless tobacco** | **Smoking tobacco** |
| --- | --- |
| **a. The questions ask about your use of smokeless tobacco (such as betel quid with tobacco, chewing tobacco leaf/ any local name……………………….), snuff, or other smokeless tobacco preparation.** | **a. The questions ask about your use of smoking tobacco (such as Cigarettes, Churoots, Pipes, Cigars, Hukkah etc.** |
| 1. Have you ever tried or experimented with using any form of smokeless tobacco products? | 1. Have you ever tried or experimented with any form of smoking tobacco products? |
| ☐ Yes  ☐ No | ☐ Yes  ☐ No |
| (If the answer is ‘No’, please go to question 14 and answer question 14 and 15)  (If the answer is ‘yes’, please answer Q: 1.1 or 1.2, 2, 3, 4. and 13) and don’t fill the question 14 and 15) | |
| 1.1. If yes, which form/product (please tick the box below that you tried, you can tick more than one) (country specific; e.g. chewing tobacco, Betel quid (pan) with tobacco, tobacco leaf or tobacco leaf mixture (Zarda), Dry tobacco powder (Gul, Mishri), khaini or tobacco lime mixture, gutka or tobacco, betel-nut and catechu mixture, Tobacco paste (Creamy snuff, Gudakhu), Snuff, pan masala, Dip, other form of ST | 1.1. If yes, which form/product (please tic the box below that you use, you can tick more than one) (country specific; modify examples for country as needed).  Cigarettes ⎯ Manufactured cigarettes, Hand-rolled cigarettes, Kretek cigarettes.    Other types of smoked tobacco: ⎯ Pipes, Cigars, mini cigars/cigarillos, Waterpipes/hookah/shisha/ narguileh/hubble-bubble, Bidis |
| \| Name and picture \| Name and picture \| Name and picture \| \| --- \| --- \| --- \| \| Name and picture \| Name and picture \| Name and picture \| | \| Name and picture \| Name and picture \| Name and picture \| \| --- \| --- \| --- \| \| Name and picture \| Name and picture \| Name and picture \| |
| ☐ 1.2. Any others in Q1.1?  Please specify…………………. | ☐ 1.2. Any others in Q1.1 ?  Please specify…………………. |
| 2. How old were you when you first tried using smokeless tobacco? | 2.How old were you when you first tried to smoke tobacco? |
| ☐ 7 years old or younger  ☐ 8 to 9 years old  ☐ 10 to 11 years old  ☐ 12 to 13 years old  ☐ 14 to 15 years old | ☐ 7 years old or younger  ☐ 8 to 9 years old  ☐ 10 to 11 years old  ☐ 12 to 13 years old  ☐ 14 to 15 years old |
| 3. Did you try any smokeless tobacco product for the first time during the last 12 months? | 3. Did you try smoking tobacco for the first time during the last 12 months? |
| ☐ Yes  ☐ No | ☐ Yes  ☐ No |
| 4. During the past 30 days, did you use any form of smokeless tobacco products? | 4.During the past 30 days, did you use any form of smoking tobacco products? |
| ☐ Yes  ☐ No | ☐ Yes  ☐ No |
| (If Yes to Q4, then answer Question 5-12; if no, then go to Question 13) | |
| 5. During the past 30 days, on how many days did you use smokeless tobacco? | 5.During the past 30 days, on how many days did you smoke tobacco? |
| ☐ 1 to 2 days  ☐ 3 to 5 days  ☐ 6 to 9 days  ☐ 10 to 19 days  ☐ 20 to 29 days  ☐ All 30 days | ☐ 1 to 2 days  ☐ 3 to 5 days  ☐ 6 to 9 days  ☐ 10 to 19 days  ☐ 20 to 29 days  ☐ All 30 days |
| 6.During the past 30 days, what smokeless tobacco brand did you usually use? | 6.During the past 30 days, what smoking tobacco brand did you usually use? |
| \| Name and picture \| Name and picture \| Name and picture \| \| --- \| --- \| --- \| \| Name and picture \| Name and picture \| Name and picture \| | \| Name and picture \| Name and picture \| Name and picture \| \| --- \| --- \| --- \| \| Name and picture \| Name and picture \| Name and picture \| |
| ☐ 6.1. Any others in Q6?  Please specify (write response here)…………………. | ☐ 6.1. Any others in Q6?  Please specify (write response here)…………………. |
| 7. Please think about the days you used smokeless tobacco during the past 30 days.  -How many times did you usually use smokeless tobacco per day? | 7. Please think about the days you smoked tobacco during the past 30 days.  -How many times did you usually smoked tobacco per day? |
| ☐ Less than once per day  ☐ Once per day  ☐ 2 to 5 times per day  ☐ 6 to 10 times per day  ☐ 11 to 20 times per day  ☐ More than 20 times per day | ☐ Less than once per day  ☐ Once per day  ☐ 2 to 5 times per day  ☐ 6 to 10 times per day  ☐ 11 to 20 times per day  ☐ More than 20 times per day |
| 8. How did you usually get smokeless tobacco that you use? | 8. How did you usually get smoking tobacco that you use? |
| ☐ I bought them in a store or a shop  ☐ I bought them from a street vendor/ temporary stall  ☐ I gave someone else money to buy them for me  ☐ I got them from someone else for free  ☐ I got them from someone else in exchange formoney  ☐ I took it without permission or knowledge  ☐ I got them some other way | ☐ I bought them in a store or a shop  ☐ I bought them from a street vendor/ temporary stall  ☐ I gave someone else money to buy them for me  ☐ I got them from someone else for free  ☐ I got them from someone else in exchange for money  ☐ I took it without permission or knowledge  ☐ I got them some other way |
| 9. Do you ever feel like using smokeless tobacco first thing in the morning? | 9. Do you ever feel like smoking tobacco first thing in the morning? |
| ☐ No, I don’t feel like using smokeless tobacco first thing in the morning  ☐ Yes, I sometimes use or feel like using smokeless tobacco first thing in the morning  ☐ Yes, I always use or feel like using smokeless tobacco first thing in the morning | ☐ No, I don’t feel like smoking tobacco first thing in the morning  ☐ Yes, I sometimes use or feel like smoking tobacco first thing in the morning  ☐ Yes, I always use or feel like smoking tobacco first thing in the morning |
| 10. How soon after you use smokeless tobacco do you start to feel a strong desire to use smokeless tobacco again that is hard to ignore? | 10. How soon after you smoked tobacco do you start to feel a strong desire to use smoke tobacco again that is hard to ignore? |
| ☐ I never feel a strong desire to use it again after using smokeless tobacco  ☐ Within 60 minutes  ☐ 1 to 2 hours  ☐ More than 2 hours to 4 hours  ☐ More than 4 hours but less than one full day  ☐ 1 to 3 days  ☐ 4 days or more | ☐ I never feel a strong desire to smoke again after smoking tobacco  ☐ Within 60 minutes  ☐ 1 to 2 hours  ☐ More than 2 hours to 4 hours  ☐ More than 4 hours but less than one full day  ☐ 1 to 3 days  ☐ 4 days or more |
| 11. Please rate your addiction to smokeless tobacco using the following scale *(make a circle around the appropriate number)*  *In this section of questions there are some statements related to your smokeless tobacco addiction. You have to choose the responses as per your perception from the numbers 1 to 7 where: 1 means/ 1 relates to - If you feel that you are not addicted at all to tobacco then choose this option. 2 means: if you feel you have little bit addiction for tobacco. It will keep increasing up to 7, where 7 means extremely addicted to smokeless tobacco.* | 11. Please rate your addiction to smoking tobacco using the following scale *(make a circle around the appropriate number)*  *In this section of questions there are some statements related to your smokeless tobacco addiction. You have to choose the responses as per your perception from the numbers 1 to 7 where: 1 means/ 1 relates to - If you feel that you are not addicted at all to tobacco then choose this option. 2 means: if you feel you have little bit addiction for tobacco. it will keep increasing up to 7, where 7 means extremely addicted to smokeless tobacco.* |
|  |  |
| **1 2 3 4 5 6 7**  1. I am not addicted to smokeless tobacco at all  7. I am extremely addicted to smokeless tobacco | **1 2 3 4 5 6 7**  1. I am not addicted to smoking tobacco at all  7. I am extremely addicted to smoking tobacco |
| 12. Did you ever try to stop using smokeless tobacco? | 12. Did you ever try to stop smoking tobacco? |
| ☐ Yes  ☐ No | ☐ Yes  ☐ No |
| 13. Have you ever received help or advice to help you stop using smokeless tobacco? (Select only one response) (GYTS) | 13. Have you ever received help or advice to help you stop smoking? (Select only one response) (GYTS) |
| ☐Yes, from a program or professional  ☐Yes, from friend or family member  ☐Yes, from both programs or professionals and from friends or family members  ☐ No | ☐Yes, from a program or professional  ☐Yes, from friend or family member  ☐Yes, from both programs or professionals and from friends or family members  ☐ No |
| (The following Questions 14- 15 are for non-triers only, who answered No to Q.1) | (The following Questions 14- 15 are for non-triers only, who answered No to Q.1) |
| 14. At any time during the next 12 months do you think you will use any form of smokeless tobacco? (Question for the non-user only) *(Source: GYTS, Canadian student tobacco, alcohol and drug survey 2017)* | 14. At any time during the next 12 months do you think you will smoke tobacco? |
| ☐ Definitely not  ☐ Probably not  ☐ Probably yes  ☐ Definitely yes | ☐ Definitely not  ☐ Probably not  ☐ Probably yes  ☐ Definitely yes |
| **(if the answer is ‘Definitely not or probably not’ in Q14 then go to Q 15. If the answer is ‘Definitely yes’ or probably yes’, do Q14.1)** | **(if the answer is ‘Definitely not or probably not’ in Q14 then go to Q 15. If the answer is ‘Definitely yes’ or probably yes’, do Q14.1)** |
| **14.1 Why will you consider using smokeless tobacco products? (You can tick more than one option)** | **14.1 Why will you consider smoking tobacco products? (You can tick more than one option)** |
| ☐ Personal enjoyment  ☐ Out of curiosity  ☐ For the sake of experiencing how it feels  ☐ Improve body image  ☐ Price is lower  ☐ My family members use these  ☐ My friends use these  ☐ Acceptable at my home/ family level  ☐ Acceptable at the society  ☐ Other……………………………  14.2. Any others in Q14.1,  please specify (write response here)-------- | ☐ Personal enjoyment  ☐ Out of curiosity  ☐ For the sake of experiencing how it feels  ☐ Improve body image  ☐ Price is lower  ☐ My family members use these  ☐ My friends use these  ☐ Acceptable at my home/ family level  ☐ Acceptable at the society  ☐ Other……………………………  14.2. Any others in Q14.1,  please specify (write response here)----------- |
| 15. If one of your friends offered you a smokeless tobacco product, would you use it? | 15. If one of your friends offered you a smoking tobacco product, would you use it? |
| ☐ Definitely not  ☐ Probably not  ☐ Probably yes  ☐ Definitely yes | ☐ Definitely not  ☐ Probably not  ☐ Probably yes  ☐ Definitely yes |
| **b. Smokeless tobacco expectation scale**  for all (both user and non-user) | **b. Smoking tobacco expectation scale**  for all (both smoker and non-smoker) |
| Please respond to the following statements, regarding how likely the following is to occur from using smokeless tobacco. (Examples of smokeless tobacco include the following: List will be shown)  You are expected to respond by writing the number from scale (0 to 9) in front of each statement, where  *1 means: very unlikely, if you think that the items are very unlikely to occur by using ST, 2 means: if you think that it is little bit likely to occur by using ST. It will keep increasing up to 9, where 9 means ‘it is very likely to occur by using ST’.* | Please respond to the following statements, regarding how likely the following is to occur from smoking tobacco. (Examples of smoking tobacco include the following: List will be shown)  You are expected to respond by writing the number from scale (0 to 9) in front of each statement, where  *1 means: very unlikely, if you think that the items are very unlikely to occur by using Smoking, 2 means: if you think that it is little bit likely to occur by smoking. It will keep increasing up to 9, where 9 means ‘it is very likely to occur by smoking’.* |
| 0 1 2 3 4 5 6 7 8 9  1. Very 9. Very likely  Unlikely | 0 1 2 3 4 5 6 7 8 9  1. Very 9. Very likely  Unlikely |
| - 1. Smokeless tobacco will lead to gum disease_ | - 1. Smoking tobacco will lead to gum disease_ |
| 1.2. Smokeless tobacco leads to nicotine (one component of tobacco) addiction____ | 1.2. Smoking tobacco leads to nicotine (one component of tobacco) addiction_____ |
| 1.3. Smokeless tobacco negatively impacts your health_ | 1.3. Smoking tobacco negatively impacts your health__ |
| 1.4. Smokeless tobacco leads to discolored teeth_____ | 1.4. Smoking tobacco leads to discolored teeth______ |
| 1.5. Smokeless tobacco causes tooth decay________ | 1.5. Smoking tobacco causes tooth decay________ |
| 1.6. Smokeless tobacco use makes feel content_____ | 1.6. Smoking tobacco use makes feel content_______ |
| 1.7. Smokeless tobacco causes time to go by faster____ | 1.7. Smoking tobacco causes time to go by faster____ |
| 1.8. Smokeless tobacco use gives energy_______ | 1.8. Smoking tobacco use gives energy_______ |
| 1.9. Smokeless tobacco use allows being more focused_ | 1.9. Smoking tobacco use allows being more focused_ |
| 1.10. Smokeless tobacco use keeps me busy_______ | 1.10. Smoking tobacco use keeps me busy_______ |
| 2. Once someone has started using smokeless tobacco, do you think it would be difficult for them to quit? | 2. Once someone has started smoking tobacco, do you think it would be difficult for them to quit? |
| ☐ Definitely not  ☐ Probably not  ☐ Probably yes  ☐ Definitely yes | ☐ Definitely not  ☐ Probably not  ☐ Probably yes  ☐ Definitely yes |
| 3. Do you think Smokeless tobacco helps people to feel more comfortable or less comfortable at celebrations, parties, or in other social gatherings? | 3. Do you think Smoking tobacco helps people to feel more comfortable or less comfortable at celebrations, parties, or in other social gatherings? |
| ☐more comfortable  ☐less comfortable  ☐No difference whether using or not | ☐more comfortable  ☐less comfortable  ☐No difference whether using or not |
| 4. Based on what you know or believe, does smokeless tobacco cause cancer? | 4. Based on what you know or believe, does smoking tobacco cause cancer? |
| ☐ Yes  ☐ No  ☐ Don't know | ☐ Yes  ☐ No  ☐ Don't know |
| 5. What is the condition of your oral health?  ☐ Good  ☐ moderate  ☐ poor  6. How happy are you with your oral health condition?  ☐ Happy  ☐ neither happy nor unhappy  ☐ unhappy | |
| **c. Packaging and labelling of tobacco products** | |
| **Smokeless tobacco** | **Smoking tobacco** |
| 1. During the past 12 months, how often have you noticed: smokeless tobacco product packages carry large health warnings and/or messages describing the harmful effects of tobacco use? | 1. During the past 12 months, how often have you noticed: smoking tobacco product packages carry large health warnings and/or messages describing the harmful effects of tobacco use? |
| ☐ Never  ☐ Rarely  ☐ Sometimes  ☐ Often  ☐ Very often  ☐ Can not recall/ not sure | ☐ Never  ☐ Rarely  ☐ Sometimes  ☐ Often  ☐ Very often  ☐ Can not recall/ not sure |
| (If your answer is ‘Never or Can not recall/ not sure’ please go to question 4; if your answer: Rarely/Sometimes/Often/ Very often then answer Q 2, 3, and then 4) | |
| 2.In which form was it? (you can choose more than one) | 2.In which form was it? (you can choose more than one) |
| ☐ a) Pictorial/ graphic  ☐ b) Text  ☐ c) both picture and text | ☐ a) Pictorial/ graphic  ☐ b) Text  ☐ c) both picture and text |
| (If b or c) is chosen in Q2, do Q2.1)  2.1 If the text was there, what was the language of the warning? | (If b or c) is chosen in Q2, do Q2.1)  2.1 If the text was there, what was the language of the warning? |
| ☐ a) Bengali /Hindi/Urdu (Country specific)  ☐ b) English  ☐ c) Other language……  ☐ d) I have not noticed | ☐ a) Bengali /Hindi/Urdu (Country specific)  ☐ b) English  ☐ c) Other language……  ☐ d) I have not noticed |
| 3. What you think about the health warnings on smokeless tobacco packages? | 3. What you think about the health warnings on smoking tobacco packages? |
| ☐ I saw but I didn’t think much of them  ☐ I saw and that led me to think about not to start using smokeless tobacco. (applicable for those who never tried smokeless tobacco, if you select ‘no’ for Q1 in section A)  ☐ I saw and they led me to think about quitting use of smokeless tobacco. (applicable for those who ever tried smokeless tobacco, if you select ‘yes’ for Q1 in section A) | ☐ I saw but I didn’t think much of them  ☐ I saw and that led me to think about not to start smoking tobacco. (applicable for those who never smoked, if you select ‘no’ for Q1 in section A)  ☐ I saw and they led me to think about quitting smoking tobacco. (applicable for those who ever tried smoking, if you select ‘yes’ for Q1 in section A) |
| 4.In the last 12 month, have you seen any smokeless tobacco product/ packets being openly displayed, including on shelves or on the counter? By openly displayed, I mean without any shutters or screens covering the packs. | 4.In the last 12 month, have you seen any smoking tobacco product/ packets being openly displayed, including on shelves or on the counter? By openly displayed, I mean without any shutters or screens covering the packs. |
| ☐ Never  ☐ Rarely  ☐ Sometimes  ☐ Often  ☐ Very often  ☐ Can not recall/ not sure | ☐ Never  ☐ Rarely  ☐ Sometimes  ☐ Often  ☐ Very often  ☐ Can not recall/ not sure |
| d. The next questions ask about promotion against using smokeless tobacco or smoking tobacco, education, communication, training, awareness | |
| **Smokeless tobacco** | **Smoking tobacco** |
| 1.During the past 12 months, how frequently have you noticed things (family, friends, school, media, any other) that talk about the dangers of using smokeless tobacco? | 1.During the past 12 months, how frequently have you noticed things (family, friends, school, media, any other) that talk about the dangers of using smoking tobacco? |
| ☐ Never  ☐ Yes, rarely  ☐Yes, sometimes  ☐Yes, often  ☐Yes, very often | ☐ Never  ☐ Yes, rarely  ☐Yes, sometimes  ☐Yes, often  ☐Yes, very often |
| - 1. (If the answer is Yes in Q1, then answer Q1.1. If you answer ‘Never’ then go to Q 2)   Where did you find this information? (You can choose more than one) | - 1. (If the answer is Yes in Q1, then answer Q1.1. If you answer ‘Never’ then go to Q 2)   Where did you find this information? (You can choose more than one) |
| ☐ a) Packet of the product ☐ i) Bill board  ☐ b) Newspaper ☐ j) Books  ☐ c) Magazines/journal ☐ k) Radio  ☐ d) TV ☐ l) Movies  ☐ e) Internet ☐ m) in the shop  ☐ f) school ☐ n) family  ☐ g) friends ☐ o) Relatives  ☐ h) Dentist/doctor/ Health worker  ☐ p) other…………………………. | ☐ a) Packet of the product ☐ i) Bill board  ☐ b) Newspaper ☐j) books  ☐ c) Magazines/journal ☐ k) Radio  ☐ d) TV ☐ l) Movies  ☐ e) Internet ☐ m) in the shop  ☐ f) school ☐ n) family  ☐ g) friends ☐ o) Relatives  ☐ h) Dentist/doctor/ Health worker  ☐ p) other…………………………. |
| 2. During the past 12 months, did you see or hear any anti-smokeless tobacco use messages at sports events, fairs, concerts, or community events, or social gatherings? | 2. During the past 12 months, did you see or hear any anti-smoking tobacco use messages at sports events, fairs, concerts, or community events, or social gatherings? |
| ☐ Yes  ☐ No  ☐ I did not go to sports events, fairs, concerts, or community events, or social gatherings. | ☐ Yes  ☐ No  ☐ I did not go to sports events, fairs, concerts, or community events, or social gatherings. |
| 3. During the past 12 months, were you taught in any of your classes about the dangers of use of smokeless tobacco? | 3. During the past 12 months, were you taught in any of your classes about the dangers of smoking tobacco? |
| ☐ Yes  ☐ No  ☐ I don’t know | ☐ Yes  ☐ No  ☐ I don’t know |
| 4. What do you think about any form of anti smokeless tobacco campaign for raising public awareness? | 4. What do you think about any form of anti tobacco smoking campaign for raising public awareness? |
| ☐ Not applicable (I’ve never seen any)  ☐ I saw but I didn’t think much of them  ☐I saw and that lead me to think about not to start using smokeless tobacco (applicable for those who never tried smokeless tobacco,if you select ‘no’ for Q1 in section A)  ☐I saw and that they led me to think about quitting use of smokeless tobacco (applicable for those who ever tried smokeless tobacco, if you select ‘yes’ for Q1 in section A) | ☐ Not applicable (I’ve never seen any.)  ☐ I saw but I didn’t think much of them  ☐I saw and that lead me to think about not to start smoking tobacco (applicable for those who never smoked, if you select ‘no’ for Q1 in section A)  ☐I saw and that they led me to think about quitting smoking (applicable for those who ever tried smoking, if you select ‘yes’ for Q1 in section A) |
| **e.** **The next questions ask about banning advertisements or promotions for tobacco (smokeless tobacco /cigarettes and other smoked tobacco).** | |
| **Smokeless tobacco** | **Smoking tobacco** |
| 1. During past 12 months, how often have you noticed things that promote smokeless tobacco? | 1. During past 12 months, how often have you noticed things that promote smoking tobacco? |
| ☐ Never  ☐ Yes, rarely  ☐ Yes, sometimes  ☐ Yes, often  ☐ Yes, very often | ☐ Never  ☐ Yes, rarely  ☐ Yes, sometimes  ☐ Yes, often  ☐ Yes, very often |
| (If the answer in Q1 is yes, then answer Q1.1. If you answer ‘Never’ then go to Q 2) | (If the answer in Q1 is yes, then answer Q1.1. If you answer ‘Never’ then go to Q 2) |
| - 1. Where did you see these promotions? (You can choose more than one option) | - 1. Where did you see these promotions? (You can choose more than one option) |
| ☐ a) TV advertisement ☐ e) Tobacco shop  ☐ b) Tobacco packet ☐ f) Tobacco company  ☐ c) Family members ☐g) friends  ☐ d) Poster ☐ h) other……… | ☐ a) TV advertisement ☐ e) Tobacco shop  ☐ b) Tobacco packet ☐ f) Tobacco company  ☐ c) Family members ☐ g) Friends  ☐ d) Poster ☐ h) Other……… |
| 2.During the past 12 months, did you see any people using smokeless tobacco on TV, in videos, or in movies? | 2.During the past 12 months, did you see any people smoking tobacco on TV, in videos, or in movies? |
| ☐ Yes  ☐ No  ☐ I did not watch TV, videos, or movies in the past 12 months | ☐ Yes  ☐ No  ☐ I did not watch TV, videos, or movies in the past 12 months |
| 3. During the past 12 months, did you see any advertisements or promotions for smokeless tobacco products at points of sale (such as: street vendors, tea shops, restaurants, stores, shopping malls)? | 3. During the past 12 months, did you see any advertisements or promotions for smoking tobacco products at points of sale (such as: street vendors, tea shops, restaurants, stores, shopping malls)? |
| ☐ Yes  ☐ No  ☐ I did not visit any points of sale in the past 12 months | ☐ Yes  ☐ No  ☐ I did not visit any points of sale in the past 12 months |
| 4.Would you ever use or wear something that has a smokeless tobacco company or product name or picture on it such as a lighter, t-shirt, hat, or sunglasses? | 4.Would you ever use or wear something that has a smoking tobacco company or product name or picture on it such as a lighter, t-shirt, hat, or sunglasses? |
| ☐ Yes  ☐ No | ☐ Yes  ☐ No |
| 5.Do you have something (for example, t-shirt, pen, backpack) with a smokeless tobacco product brand logo on it? | 5.Do you have something (for example, t-shirt, pen, backpack) with a smoking tobacco product brand logo on it? |
| ☐ Yes  ☐ No | ☐ Yes  ☐ No |
| 6.During the past 12 months, if you saw any advertisements or promotions for smokeless tobacco how did this influence you? | 6.During the past 12 months, if you saw any advertisements or promotions for smoking tobacco how did this influence you? |
| ☐ I saw but I didn’t think much of them  ☐ I saw and that led me to think about start using smokeless tobacco (applicable for those who never tried smokeless tobacco,if you select ‘no’ for Q1 in section A)  ☐ I saw and that led me to think about continue using smokeless tobacco (applicable for those who ever tried smokeless tobacco, if you select ‘yes’ for Q1 in section A) | ☐ I saw but I didn’t think much of them  ☐ I saw and that led me to think about start using smoking tobacco (applicable for those who never smoked, if you select ‘no’ for Q1 in section A)  ☐ I saw and that led me to think about continue using smoking tobacco (applicable for those who ever tried smoking, if you select ‘yes’ for Q1 in section A) |
| 7.Do you think, on the whole, people are encouraged to use smokeless tobacco these days? | 7.Do you think, on the whole, people are encouraged to smoking tobacco these days? |
| ☐ Yes  ☐ No | ☐ Yes  ☐ No |
| **f. The next questions ask about your buying of smokeless/smoking tobacco products** | |
| **Smokeless tobacco** | **Smoking tobacco** |
| 1.Have you ever tried to buy smokeless tobacco products? | 1.Have you ever tried to buy smoking tobacco products? |
| ☐ Never, I did not try to buy any smokeless tobacco product  ☐ Yes, for my own use  ☐ Yes, for other people (family member, friend/ other), I do not use smokeless tobacco.  ☐ Yes, for me and other people (family member, friend/ other), I use smokeless tobacco. | ☐ Never, I did not try to buy any smoking tobacco product  ☐ Yes, for my own use  ☐ Yes, for other people (family member, friend/ other), I do not use smoking tobacco.  ☐ Yes, for me and other people (family member, friend/ other), I use smoking tobacco. |
| (If the answer is ‘never’ in Q1, please go to question 5 and 6. If you answer ‘yes’ then answer Q 2-4 and then go to question 5 and 6) | (If the answer is ‘never’ in Q1, please go to question 5 and 6. If you answer ‘yes’ then answer Q 2-4 and then go to question 5 and 6) |
| 2. Did anyone ever refuse to sell you- any smokeless tobacco product because of your young age? | 2. Did anyone ever refuse to sell you- any smoking tobacco product because of your young age? |
| ☐ Yes, someone refused to sell me smokeless tobacco product because of my age  ☐ No, no one refused to sell me smokeless tobacco product because of my age | ☐ Yes, someone refused to sell me smoking tobacco product because of my age  ☐ No, no one refused to sell me smoking tobacco product because of my age |
| 3. On the whole, do you find it easy or difficult to buy smokeless tobacco product from a shop? | 3. On the whole, do you find it easy or difficult to buy smoking tobacco product from a shop? |
| ☐ Very difficult  ☐ Fairly difficult  ☐Fairly easy  ☐Very easy  ☐Sometimes difficult, sometimes easy | ☐ Very difficult  ☐ Fairly difficult  ☐Fairly easy  ☐Very easy  ☐Sometimes difficult, sometimes easy |
| 4.Can you purchase any form of smokeless tobacco products near your school? | 4.Can you purchase any form of smoking tobacco products near your school? |
| ☐ Yes  ☐ No | ☐ Yes  ☐ No |
| 5.Approximately how many shops that sell smokeless tobacco products are there in your neighbourhood (within 5 minutes walking distance of your school)? | 5.Approximately how many shops that sell smoking tobacco products are there in your neighbourhood (within 5 minutes walking distance of your school)? |
| Please write number in letter__________________ | Please write number in letter__________________ |
| 6.Approximately how many shops that sell smokeless tobacco products are in your neighbourhood (within 5 minutes walking distance of your home)? | 6.Approximately how many shops that sell smoking tobacco products are in your neighbourhood (within 5 minutes walking distance of your home)? |
| Please write number in letter___________________ | Please write number in letter__________________ |
| **g. Tobacco price** | |
| 1.During an average day, how much money (pocket money) do you have that you can spend on yourself, whatever you want? | |
| ☐ I usually don’t have any spending money  ☐ Less than 20 (currency/day)  ☐ 20-40 (currency/day)  ☐ 40-60 (currency/day)  ☐ 60-80 (currency/day)  ☐ 80-100 (currency/day)  ☐ More than 100 (currency/day) | |
| 2.On average how much money do you pay to buy your school tiffin?/ snacks and drinks? (chocolate, biscuit, crisp, juice, fizzy drinks etc.) | |
| ☐ Not applicable (non buyer)  ☐ I usually don’t have any spending money  ☐ Less than 20 (currency/day)  ☐ 20-40 (currency/day)  ☐ 40-60 (currency/day)  ☐ 60-80 (currency/day)  ☐ 80-100 (currency/day)  ☐ More than 100 (currency/day) | |
| **Smokeless tobacco** | **Smoking tobacco** |
| 3.On average, how much do you think you spent in a day on buying smokeless tobacco for your use? | 3.On average, how much do you think you spent in a day on buying smoking tobacco for your use? |
| ☐ Not applicable (for non-trier, if you select ‘no’ for Q1 in section A)  ☐ I usually don’t have any spending money  ☐ I don’t buy smokeless tobacco product for my use  ☐ Less than 20 (currency/day)  ☐ 20-40 (currency/day)  ☐ 40-60 (currency/day)  ☐ 60-80 (currency/day)  ☐ 80-100 (currency/day)  ☐ More than 100 (currency/day) | ☐ Not applicable (for nonsmoker, if you select ‘no’ for Q1 in section A)  ☐ I usually don’t have any spending money  ☐ I don’t buy smoking tobacco product for my use  ☐ Less than 20 (currency/day)  ☐ 20-40 (currency/day)  ☐ 40-60 (currency/day)  ☐ 60-80 (currency/day)  ☐ 80-100 (currency/day)  ☐ More than 100 (currency/day) |
| **h. Exposure to smokeless tobacco/ smoking tobacco products** | |
| **Smokeless tobacco** | **Smoking tobacco** |
| 1. Do your parents/ any other family member use smokeless tobacco? | 1. Do your parents/ any other family member smoke tobacco? |
| ☐ None  ☐ both parents  ☐ Father only  ☐ Mother only  ☐ Other family member…………….. | ☐ None  ☐ both parents  ☐ Father only  ☐ Mother only  ☐ Other family member…………….. |
| 2. Which of the following best describes using smokeless tobacco inside your home? | 2. Which of the following best describes smoking tobacco inside your home? |
| ☐ Use of smokeless tobacco is not allowed in any rooms  ☐ Allowed in some rooms  ☐ Use of smokeless tobacco is not allowed in front of children  ☐ Use of smokeless tobacco is not allowed in front of elders  ☐ Allowed at home (No rules/ restriction) | ☐ Use of smoking tobacco is not allowed in any rooms  ☐ Allowed in some rooms  ☐ Use of smoking tobacco is not allowed in front of children  ☐ Use of smoking tobacco is not allowed in front of elders  ☐ Allowed at home (No rules/ restriction) |
| 3. How would your parents (mother, father or carer) react if they see you using smokeless tobacco**?** | 3. How would your parents (mother, father or carer) react if they see you smoking tobacco**?** |
| ☐no reaction  ☐will be told not to use it without anger  ☐told not to use with anger  ☐told not to use it with punishment.  ☐ Don’t know | ☐no reaction  ☐will be told not to use it without anger  ☐told not to use with anger  ☐told not to use it with punishment.  ☐ Don’t know |
| 4. During the past 7 days, on how many days has anyone used smokeless tobacco inside your home, in your presence? | 4. During the past 7 days, on how many days has anyone smoked tobacco inside your home, in your presence? |
| ☐ 0 days  ☐ 1 to 2 days  ☐ 3 to 4 days  ☐ 5 to 6 days  ☐ 7 days | ☐ 0 days  ☐ 1 to 2 days  ☐ 3 to 4 days  ☐ 5 to 6 days  ☐ 7 days |
| 5. During the past 7 days, on how many days has anyone used smokeless tobacco in your presence, inside any public place, other than your home (such as: restaurants, buses, other vehicles, trains, sports centres, shopping malls, movie theatres)? | 5. During the past 7 days, on how many days has anyone smoked tobacco in your presence, inside any public place, other than your home (such as: restaurants, buses, other vehicles, trains, sports centres, shopping malls, movie theatres)? |
| ☐ 0 days  ☐ 1 to 2 days  ☐ 3 to 4 days  ☐ 5 to 6 days  ☐ 7 days | ☐ 0 days  ☐ 1 to 2 days  ☐ 3 to 4 days  ☐ 5 to 6 days  ☐ 7 days |
| 6. During the past 7 days, on how many days has anyone used smokeless tobacco in your presence, at any outdoor public place (such as: playgrounds, bus- stops, parks)? | 6. During the past 7 days, on how many days has anyone used smoked tobacco in your presence, at any outdoor public place (such as: playgrounds, bus- stops, parks)? |
| ☐ 0 days  ☐ 1 to 2 days  ☐ 3 to 4 days  ☐ 5 to 6 days  ☐ 7 days | ☐ 0 days  ☐ 1 to 2 days  ☐ 3 to 4 days  ☐ 5 to 6 days  ☐ 7 days |
| 7. During the past 7 days, on how many days did you see anyone using smokeless tobacco inside the school building or outside on school property? | 7. During the past 7 days, on how many days did you see anyone smoked tobacco inside the school building or outside on school property? |
| ☐ 0 days  ☐ 1 to 2 days  ☐ 3 to 4 days  ☐ 5 to 6 days  ☐ 7 days | ☐ 0 days  ☐ 1 to 2 days  ☐ 3 to 4 days  ☐ 5 to 6 days  ☐ 7 days |
| 8. During school hours, how often do you see teachers using smokeless tobacco in the school building/ outdoors on school premises? | 8. During school hours, how often do you see teachers smoking tobacco in the school building/ outdoors on school premises? |
| ☐ About every day  ☐ Sometimes  ☐ Never  ☐ Don’t know | ☐ About every day  ☐ Sometimes  ☐ Never  ☐ Don’t know |
| 9. Do you feel attracted to use smokeless tobacco if you see others using? | 9. Do you feel attracted to use smoking tobacco if you see others using? |
| ☐ Not applicable ( did not see others using smokeless tobacco)  ☐ I saw but I didn’t think much of them  ☐ I saw and felt attracted; that led me to think about starting to use smokeless tobacco (applicable for those who never tried smokeless tobacco, if you select ‘no’ for Q1 in section A)  ☐ I saw and felt attracted; that led me to think about continuing using smokeless tobacco (applicable for those who ever tried smokeless tobacco, if you select ‘yes’ for Q1 in section A) | ☐ Not applicable (did not see others smoking tobacco)  ☐ I saw but I didn’t think much of them  ☐ I saw and felt attracted; that led me to think about starting to smoke tobacco (applicable for nonsmoker, if you select ‘no’ for Q1 in section A)  ☐ I saw and felt attracted; that led me to think about continuing smoking tobacco (applicable for smoker, if you select ‘yes’ for Q1 in section A) |
| 10. Approximately How many of your friends use smokeless tobacco? | 10. Approximately How many of your friends smoke tobacco? |
| ☐ None of my friends use smokeless tobacco  ☐ 1 to 2  ☐ 3 to 4  ☐ 5 to 6  ☐ more than six | ☐ None of my friends smoke tobacco  ☐ 1 to 2  ☐ 3 to 4  ☐ 5 to 6  ☐ more than six |
|  | 11. Do you think the smoke from other people’s tobacco smoking is harmful to your health? |
|  | ☐ Definitely not  ☐ Probably not  ☐ Probably yes  ☐ Definitely yes |
| **(Question to non-trier only,** if you select ‘no’ for Q1 in section A) | **(Question to nonsmoker only,** if you select ‘no’ for Q1 in section A) |
| **12. Why you do not use smokeless tobacco? (You can tick more than one box)** | **12. Why you do not smoke tobacco? (You can tick more than one box)** |
| ☐ a) I do not want to/ my personal choice  ☐ b) Due to negative health effects.  ☐ c) I have health problem  ☐ d) Price is higher than I can afford  ☐ e) My friends do not use these  ☐ f) My family members/ relatives do not use these  ☐ g) Not Acceptable at my home/ family level  ☐ h) Not Acceptable in society  ☐ i) I do not know about any smokeless tobacco product. | ☐ a) I do not want to/ my personal choice  ☐ b) Due to negative health effects.  ☐ c) I have health problem  ☐ d) Price is higher than I can afford  ☐ e) My friends do not use these  ☐ f) My family members/ relatives do not use these  ☐ g) Not Acceptable at my home/ family level  ☐ h) Not Acceptable in society  ☐ i) I do not know about any smoking tobacco product. |

**Additional questions on electronic cigarettes**

Ad1. Did you ever hear about electronic cigarettes?

☐ Yes

☐ No

(if Yes in Ad1, do Ad2)

Ad2. Have you ever tried electronic cigarettes?

☐ Yes

☐ No
