# Supplementary material for: Conducting tobacco control surveys among schoolchildren in Bangladesh, India and Pakistan: A feasibility study
Source: PLOS Glob Public Health. 2024 Oct 3;4(10):e0003784. doi: 10.1371/journal.pgph.0003784 (PMC11449278; doi:10.1371/journal.pgph.0003784)
Supplement: S3 Text — (DOCX) [file pgph.0003784.s003.docx]

**Supplementary material 3: Triangulation matrix**

| **Study task (and objective)** | **Records and field notes** | **Survey data** | **IDIs with head teachers (HTs)** | **IDIs with class teachers (CTs)** | **FGDs with students** | **Conclusion** |
| --- | --- | --- | --- | --- | --- | --- |
| School selection (objective 1) | Mean 3.5 months to prepare school lists (B4, I2, P4.5). Time unrelated to number of schools (B70, I1056, P71). B and P faced challenges in accessing the information. | - | - | - | - | Lengthy task, delays due to difficulties in accessing information in B and P. |
| School recruitment (objective 1) | Recruitment rates: B88.9%, I72.7%, P90%. Securing higher authority permissions took time, mean 3.2 months (B1, I6, P3). Some reluctance in I and P.  4 schools declined usually citing time pressures, not relevant to girls (P).  Days to secure school agreement: B2.5, I 3.8, P34.3. Delays in P due to concerns about students’ young age. | - | Approved the recruitment process, some requested more details and endorsements from Government, other schools. | - | - | Good recruitment achieved and task acceptable to HTs, but lengthy process. Due to some reluctance by higher authorities in I and P and by HTs in P. |
| School retention (objective 1) | No data | - | - | - | - | Not possible to assess due to COVID-19. |
| Student selection (objective 1) | Final number of eligible students: B1261, I1045 and P770. | - | Consensus that selecting classes was relatively straightforward, (5-30 minutes), except when they consulted with CTs. Evidence that used own approaches (not per protocol). | CTs selected students in their classes using a variety of approaches (not per protocol), fast process for most (10-45 minutes). Delays due to wider consultation, other school commitments. | - | High numbers of eligible students achieved, and task generally completed quickly and easily. Deviations to protocol by HTs and CTs. |
| Student recruitment - Distributing study information (objectives 1 and 2) | - | - | Consensus that task was easy (30—60 minutes) except when eligible students absent. | Consensus that task was easy (30—60 minutes) except when eligible students absent. Some CTs in P requested researchers to do this task. | Most found information to be understandable and shared it/discussed study with their parents. Many in rural areas mentioned their parents could not read. | Task generally completed quickly and easily. |
| Student recruitment - Collecting parental consent (objectives 1 and 2) | Consent rates: B54.8%, I79.9%, P65.4%  Illiteracy amongst parents was key reason for refusal.  Multiple visits needed to collect the forms in. | - | Consensus that illiteracy amongst parents was key reason for refusal – they would not consent to something they did not fully understand.  Some HTs were telephoned by parents with questions. In I and P some parents saw the research as inappropriate for girls. Parent meeting offered as better approach. | Consensus that illiteracy amongst parents was key reason for refusal – they would not consent to something they did not fully understand.  Collecting in the forms was usually quick (20-40 minutes) but took several weeks due to student absenteeism, forgetting, holidays. Parent meeting offered as better approach | - | Low to moderate consent rates. Task was challenging due to parents’ illiteracy, and delays in collecting in forms due to students forgetting, being absent, school holidays. Parent meeting offered as a solution. |
| Student recruitment - Collecting student assent (objective 1) | Assent rates: B89.4%, I100%, P95.3%  Absent from school or forgetting were key reasons for no assent.  Most students needed help to complete the forms. | - | - | Collecting in forms took 30-60 minutes. Most students needed help to complete the forms. | Some students were hesitant to take part due to mis/lack of understanding about the study. | High assent rates. Students needed help with the forms. Some student hesitation but lack of assent typically due to being absent from school or forgetting. |
| Student retention (objective 1) | No data | - | - | - | - | Not possible to assess due to COVID-19. |
| Survey administration (objective 2) | Usually lead by research team in class time. Mean completion time: B 35.2 minutes, I 97.5 minutes, 78.3 minutes. Time not related to researcher: student ratio. | - | Majority said it was difficult to fit into school timetable, could not be completed in one school period. Some concerns in B and I about impact on lessons/tests. Suggestion to do survey at start of year/in vacations and outside of class time. | Usually led by research team in class time. Suggestion to do survey at start of year/in vacations and outside of class time. | Students enjoyed completing the survey both in/outside of class. Suggestion to do survey at start of year/in vacations and outside of class time. | Task was disruptive to the school day, taking more than 1 class period. |
| Questionnaire completion (objectives 2 and 3) | Intensive support needed, especially for younger children. Unfamiliar words were a challenge. | Completion rates: B99.8%, I82.9%, P100%  No missing data: B100%, I96.2%, P99.2%.  Behaviour questions well completed (range 99-100%). | - | View that questionnaire was too long, and concerns about language and format. | Most students enjoyed completing it. View that questionnaire was too long, some confusion with unfamiliar words, but could differentiate between two smoking behaviours. | Good completion rates and little missing data. However this required intensive support from the research team. |

*Note.* Any differences by country are noted. B=Bangladesh, I=India, P=Pakistan.
