# Supplementary material for: Conducting tobacco control surveys among schoolchildren in Bangladesh, India and Pakistan: A feasibility study
Source: PLOS Glob Public Health. 2024 Oct 3;4(10):e0003784. doi: 10.1371/journal.pgph.0003784 (PMC11449278; doi:10.1371/journal.pgph.0003784)
Supplement: S4 Text — (DOCX) [file pgph.0003784.s004.docx]

**
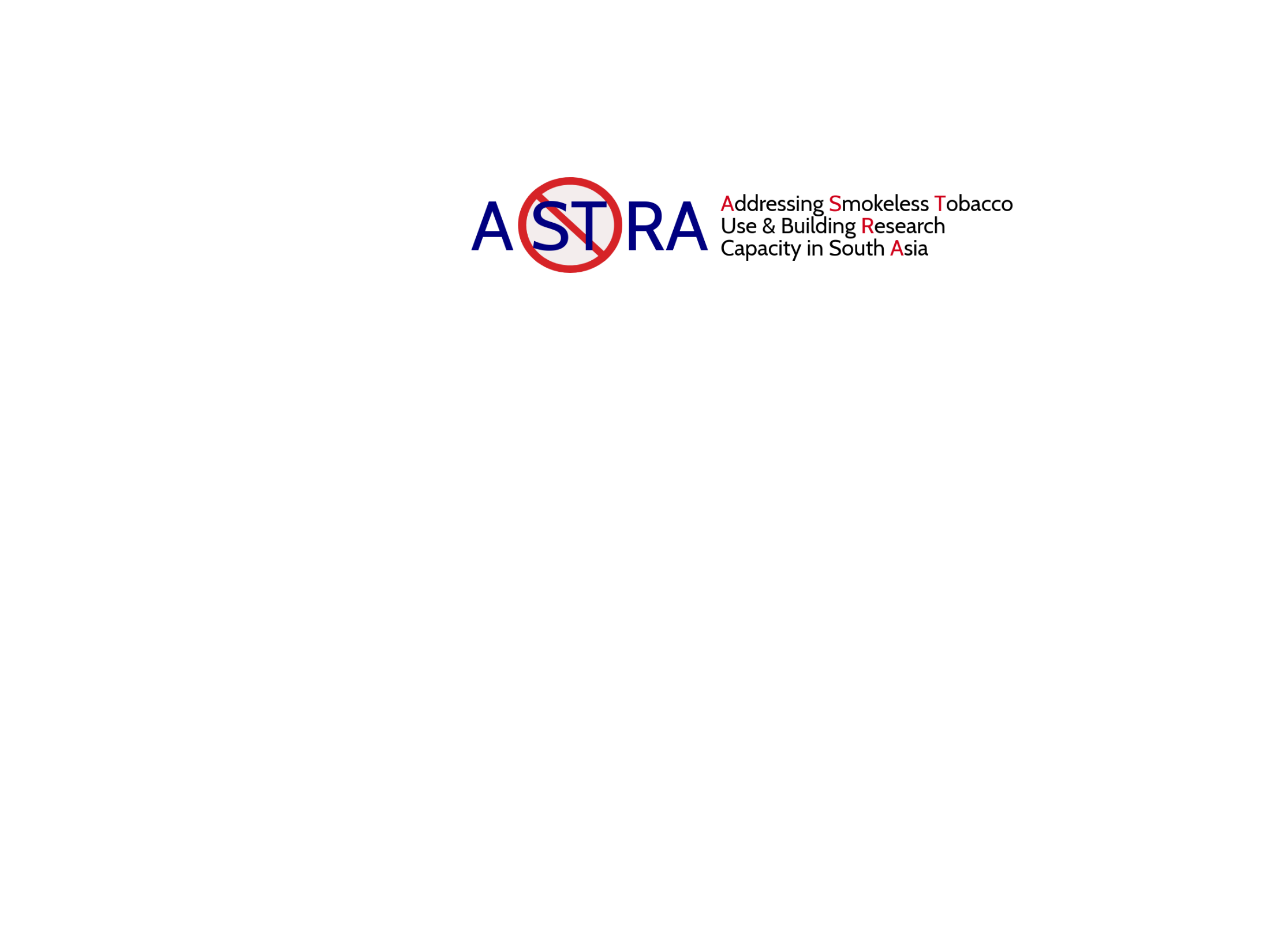
**

**Topic guide for STUDENTS – ROUND 1**

**(to be done as soon as possible after they complete the survey)**

This is to be used with students of classes 6, 7 and 8 (1 focus group per class)

*(Have these study documents with you to remind the students – student information sheet, student assent form, survey)*

| **PROCESS**  *BEFORE THE FOCUS GROUP*   - Thank the students for their time and contribution. - Tell the students that this will be like a group conversation. - It will last 20-30 minutes - Complete the Student Demographics Information Form. - Tell them, we would like to hear their experiences of taking part in our tobacco study. We will ask for their ideas on how we can improve the study ready for the second round of data collection, particularly as we are intending to run our study in the future on a larger scale with more students in more schools. - The discussion will be digitally audio-recorded. Explain why – because we can talk to each other directly, without the researcher having to write it down and more accurately record what they say. - Reassure them that there are no right or wrong answers, we are really interested in their experiences and views so that we can improve the study, so please be honest. Participation is anonymous and they will not be identified. - Tell them they can stop at any time. - Turn on the recorder and start the focus group   *AT THE END OF THE FOCUS GROUP*   - Thank the participant(s) again. |
| --- |

*If you do not have much time – just do sections 1 and 4*

| 1. **Overall how was the experience?** |
| --- |
| - What did you think about taking part in our tobacco study? - Did you enjoy it? - What did you like about it? Why did you like that? - Was there anything that you did not like? Why did you not like that? - How could we make it more enjoyable for other students? |

| 1. **Hearing about the study** |
| --- |
| - How did you hear about the study? Who from? - Did you read this information sheet? *(look together at the student information sheet)*   - Is there anything that you did not understand? What?   - Is there any information we need to change? What?   - Is there any extra information that we need to add? What? - If we do a larger study with more students in more schools, should we keep or change the way we invite those students to take part?   - How else could we do it? Why is that a better way? - How could we encourage more students to take part? |

| 1. **Agreeing to take part in the study** |
| --- |
| - Who did you discuss the study with?   - Did you talk to your parents about it? What did they say? - Why did you decide to take part? - Did you have any reservations about taking part? What were they? - Did you complete this form? *(look together at the assent form)*   - Where were you when you completed it?   - Did anyone help you? Who?   - Is there Is there anything that you did not understand? What? How should we change it? - If we do a larger study with more students in more schools, should we keep or change the way we ask students to decide to take part?   - How else could we do it? Why is that a better way? |

| 1. **Completing the questionnaire** |
| --- |
| - Where did you complete the questionnaire?   - What did you like about doing it there?   - Was there anything you did not like about doing it there?   - Where would you prefer? Why? - When did you complete the questionnaire?   - What did you think of doing it then?   - When would you prefer? A particular week in the school term? A particular day? A particular time?   - Why do you prefer then? - We asked you to complete the questionnaire on paper?   - How would you prefer to complete the questionnaire? (e.g. writing on paper, on a computer) Why? - How long did it take to complete?   - Was that too long/short/OK? Why?   - How long is best? - Was the questionnaire easy or difficult to complete? Why is that? *(look together at the questionnaire)*   - Which questions were difficult? Why was it difficult?   - What did you do when the question was difficult? - Could you see the difference between the smoking and the smokeless tobacco questions? How?   - Do we need make that difference clearer for students? How? - If we do a larger study with more students in more schools, should we keep or change how we ask them to complete the questionnaire?   - How else could we do it? (place, time, paper versus online, length of questionnaire) |

| **5 Final question** |
| --- |
| - Is there anything else about taking part in our tobacco study that you want to tell us? |
