# Supplementary material for: Conducting tobacco control surveys among schoolchildren in Bangladesh, India and Pakistan: A feasibility study
Source: PLOS Glob Public Health. 2024 Oct 3;4(10):e0003784. doi: 10.1371/journal.pgph.0003784 (PMC11449278; doi:10.1371/journal.pgph.0003784)
Supplement: S5 Text — (DOCX) [file pgph.0003784.s005.docx]

**
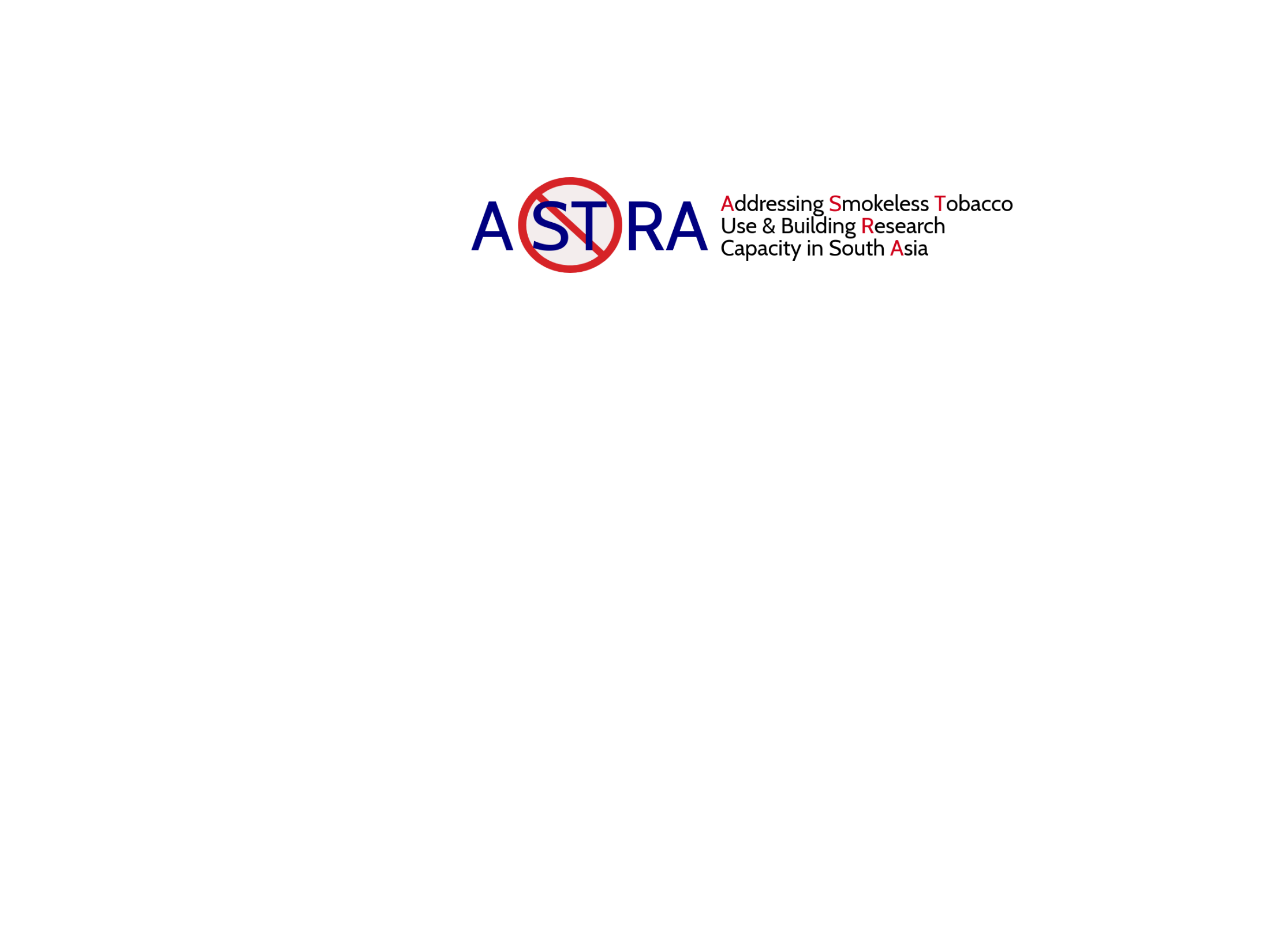
**

**Topic guide for CLASS TEACHERS**

This is to be used with the class teachers of classes 6, 7 and 8; ideally as a small group interview (or if not possible then as individual interviews)

| **PROCESS**  *BEFORE THE INTERVIEW STARTS*   - Thank the participant(s) for their time and contribution. - Explain that the duration the interview will be approximately 30-45 minutes. minutes. - Check that the participant(s) have read the Participant Information Sheet - Ask them if they have any questions about the interview and answer these. - Complete and sign consent form (2 copies, participant keeps one, researcher keeps one). - Complete the Class Teacher Demographics Information Form. - Tell the participant(s) that this will be like a group conversation. - We would like to hear their experience of their school taking part in our study. We will ask for their ideas on how we can improve the study ready for the second round of data collection, particularly as we are intending to run our study in the future on a larger scale with more students in more schools. The interview will be digitally audio-recorded. - Explain why we are using the recorder– because we can talk to each other directly, without the researcher having to write it down and more accurately record what they say. (If they refuse for the interview to be recorded, then you will need to take notes). - Reassure them that there are no right or wrong answers, we are really interested in their experiences and views so that we can improve the study, so please be honest. Participation is anonymous and they will not be identified. - Tell them they can stop at any time. - Turn on the recorder and start interview.   *AT THE END OF THE INTERVIEW*   - Thank the participant(s) again. |
| --- |

If they were not involved in some of these tasks – move onto the next task.

The first task we would like to ask you about is selecting children to take part in the study.

| **TASK 1: Selecting children**   - Was it you who did this? If not, who? - What was the procedure we asked you to use to select the children? - How long did the work associated with this task take? - How easy or difficult was this? If difficult, why was that? - Would you have preferred to select the children in a different way? How? What is advantage of your suggested approach? - Should we be recruiting students from other years as well? Why not/why those additional age groups? - If we do a larger study with more schools, should we keep or change the way we ask teachers to select children?   - How else could we do it? |
| --- |

The next task we would like to ask you about is distributing information to parents.

| **TASK 2: Distributing the information to parents**   - Was it you who did this? If not, who? - What was the procedure we asked you to follow to distribute the study information to parents? - How long did the work associated with this task take? - How easy or difficult was this? If difficult, why was that? - Would you have preferred to do this task in a different way? How? What is advantage of your suggested approach? - If we do a larger study with more schools, should we keep or change the way we ask schools to distribute information to parents?   - How else could we do it? |
| --- |

The next task we would like to ask you about is collecting permission from parents and students.

| **TASK 3: Collecting consent (parents) and assent (students)**   - Was it you who did this? If not, who? - What was the procedure we asked you to follow to collect in the consent forms from parents? - How long did the work associated with this task take you? - How much time (days) on average did the parents/guardian take to respond to the informed consent and information sheet? - How many parents/guardians (approximate proportion) visited the school to discuss the study, before they would give consent? - How many parents/guardians (approximate proportion) needed help to read through the information sheet and consent? - What were the most common questions that parents/guardians had? - How many parents/guardians (approximate proportion) refused to consent even after you explained the study to them? Why did they refuse? - Would you have preferred to collect consent from parents/guardians in a different way? How? What is advantage of your suggested approach? - What was the procedure we asked you to follow to collect in the assent forms from students? - Who did this? - How long did the work associated with this task take you? - How much time (days) on average did the students take to provide assent? - How many students (approximate proportion) needed help to understand the assent form? - What were the most common questions that students had? - How many students (approximate proportion) refused to participate even after you explained the study to them? Why did they refuse? - Would you have preferred to collect assent from students in a different way? How? What is advantage of your suggested approach? - If we do a larger study with more schools, should we keep or change the way we ask schools to collect consent and assent?   - How else could we do it? |
| --- |

The next task we would like to ask you about administering the questionnaire to the students.

| **TASK 4: Administering the questionnaire**  (have questionnaire available to look at)   - How long did the work associated with this take you? - How easy was it to fit it into the school timetable? - When in the school day did you do it? - How long on an average (in minutes) did it take students to complete the questionnaire? - If we do a larger study with more schools, should we keep or change the way we ask classes to administer the questionnaire?   - How else could we do it? - Did you look at the questionnaire? - Were there any questions that you were concerned about?   - Which ones?   - What is your concern? (Tips: wording, comprehension, time required for completing the questionnaires)   - How could we change them? - Would you like to suggest any additional questions? |
| --- |

And now our final questions.

| **Final questions**   - Overall, how would you describe your experience of helping with this study? Why do you say that? - Is there anything else you want to say? |
| --- |
