# Supplementary material for: Conducting tobacco control surveys among schoolchildren in Bangladesh, India and Pakistan: A feasibility study
Source: PLOS Glob Public Health. 2024 Oct 3;4(10):e0003784. doi: 10.1371/journal.pgph.0003784 (PMC11449278; doi:10.1371/journal.pgph.0003784)
Supplement: S6 Text — (DOCX) [file pgph.0003784.s006.docx]

**
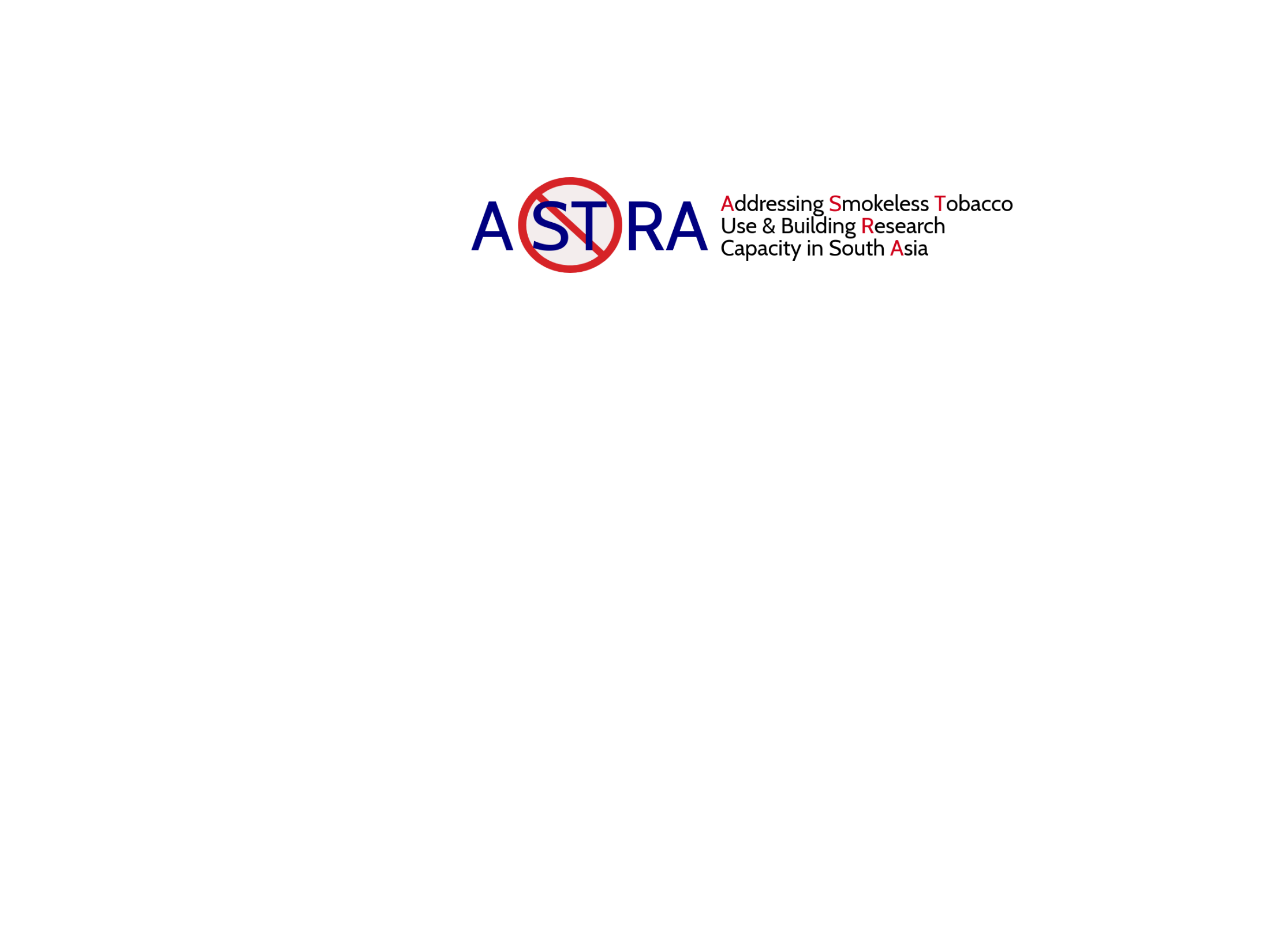
**

**School Questionnaire and Topic Guide for Head Teacher**

This is to be used with the head teacher or other representative of the school

| **PROCESS**  *BEFORE THE INTERVIEW STARTS*   - Thank the participant(s) for their time and contribution. - Explain that the duration the interview will be approximately 30 minutes. - They should have already read the Participant Information Sheet for Head Teachers and completed the consent form for Head Teachers when they agreed for the school to take part in the study. Have both documents with you, in case they have not seen them/cannot remember them/have not signed the consent form. - Ask them if they have any questions about the interview and answer these. - Complete the Head Teacher Demographics Information Form - Tell the participant that this will be like a normal conversation between 2 people. - Explain that you will first ask some questions about the school tobacco policy and whether the school canteen sells tobacco (if they have a canteen). You will write down their answers on the school questionnaire. - In the second part of the interview we would like to hear their experience of their school taking part in our study. We will also ask for their ideas on how we can improve the study ready for the second round of data collection, particularly as we are intending to run our study in the future on a larger scale with more students in more schools. This part of the interview will be digitally audio-recorded. - Explain why we are using the recorder for the second part of the interview – because we can talk to each other directly, without the researcher having to write it down and more accurately record what they say. (if they refuse for the interview to be recorded, then you will need to take notes). - Reassure them that there are no right or wrong answers, we are really interested in their experiences and views so that we can improve the study, so please be honest. Participation is anonymous and they will not be identified. - Tell them they can stop at any time. - Start the interview.   *AT THE END OF THE INTERVIEW*   - Thank the participant(s) again. |
| --- |

**Question on school tobacco policy**

**Please tick (√) the appropriate box.**

| **School policy on using smokeless tobacco** | **School policy of smoking tobacco** |
| --- | --- |
| 1. Which of the following best describes the rules about using smokeless tobacco inside the school building? | 1. Which of the following best describes the rules about people smoking tobacco inside the school building? |
| ☐ Using smokeless tobacco is not allowed anywhere inside the school building  ☐ Using smokeless tobacco is allowed inside some rooms apart from the classrooms  ☐ Using smokeless tobacco is allowed inside classrooms  ☐ There is no rule about using smokeless tobacco in school building  ☐ Other  Please explain Other:  ……………………………………………………………… | ☐ Tobacco smoking is not allowed anywhere inside the school building  ☐ Tobacco smoking is allowed inside some rooms apart from the classrooms  ☐ Tobacco smoking is allowed inside classrooms  ☐ There is no rule about tobacco smoking in school building  ☐ Other  Please explain Other:  ……………………………………………………………… |
| 2. Which of the following best describes the rules about using smokeless tobacco outside the school building (within the school premises)? | 2. Which of the following best describes the rules about tobacco smoking outside the school building (within the school premises)? |
| ☐ Using smokeless tobacco is not allowed for anyone  ☐ Using smokeless tobacco is not allowed for students  ☐ Using smokeless tobacco is allowed for anyone  ☐ There is no rule about using smokeless tobacco within school premises  ☐ Other  Please explain Other:  ……………………………………………………………… | ☐ Tobacco smoking is not allowed for anyone  ☐ Tobacco smoking is not allowed for students  ☐ Tobacco smoking is allowed for anyone  ☐ There is no rule about tobacco smoking within school premises  ☐ Other  Please explain Other:  ……………………………………………………………… |
| 3. Are your teachers allowed to use smokeless tobacco in front of students? | 3. Are your teachers allowed to smoke tobacco in front of students? |
| ☐ Yes  ☐ No | ☐ Yes  ☐ No |
| 4. Are students allowed to use smokeless tobacco in front of teachers? | 5. Are students allowed to smoke tobacco in front of teachers? |
| ☐ Yes  ☐ No | ☐ Yes  ☐ No |

Q. Is there any school canteen?

☐ Yes

☐ No

(If the answer is ‘Yes’ go to the next section. If the answer is ‘No’, go to the section on their experience of administering the tobacco survey in the school)

**Information on school canteen tobacco selling**

| **Smokeless tobacco products** | **Smoking tobacco products** |
| --- | --- |
| 1. Are any form of smokeless tobacco products available in the school canteen? | 1. Are any form of tobacco smoking products available in the school canteen? |
| ☐ Yes  ☐ No  (if ‘Yes’, go to question 2, if ‘No’ go to Question 3) | ☐ Yes  ☐ No  (if ‘Yes’, go to question 2, if ‘No’ go to Question 3) |
| 2. Does the canteen sell any smokeless tobacco products to the students? | 2. Does the canteen sell any tobacco smoking products to the students? |
| ☐ Yes  ☐ No | ☐ Yes  ☐ No |
| 3. Are your staff allowed to use smokeless tobacco inside the school canteen?  ☐ Yes  ☐ No | 3. Are your staff allowed to smoke tobacco inside the school canteen?  ☐ Yes  ☐ No |
| 4. Are the students allowed to use smokeless tobacco inside the school canteen? | 4. Are the students allowed to smoke tobacco inside the school canteen? |
| ☐ Yes  ☐ No | ☐ Yes  ☐ No |

**Experience of administering the tobacco survey in the school**

(this part of the interview needs to be digitally audio-recorded)

Now we will discuss your experience of hosting this study in your school.

The first topic we would like to ask you about how we invited you to take part in the study.

| **TOPIC 1: Invitation to take part and securing agreement**  (have letter, information sheet, consent form available to look at)  We sent you a letter with some study information to invite your school to take part in the study.   - Did you see the letter? Or did it go to someone else in the school? Who? - Who is the best person to send the invitation letter to? Why them? - When you read the letter, why did you want your school to take part in the study? - How useful was the letter? Is there anything else that it should include? - Did you then have a meeting with a member of the research team to discuss your school’s involvement?   If yes   - Who attended from your school? - How long did it last? - What did you discuss? - How useful was this meeting? Why was that? - What else should we tell headteachers in this meeting?   If no   - Why was that? - Would a meeting have been useful? Why? - Did you need to get approval from anyone else? (for example any other school authority/directorate or Board?)   - Who?   - How long did this take? - Was there anything that made this difficult? Please tell me. - Did you see this information sheet? - When did you see it? - How useful was this information? Why was that? - What else should we tell headteachers in this information sheet before the consent to take part? - If we do a larger study with more schools, should we keep or change the way we invite schools to take part?   - How else could we do it? |
| --- |

Next we would like to ask you about how you selected the classes to be included in the study.

| **TASK 2: Selecting classes**   - How long did the work associated with this task take? - How easy or difficult was this? If difficult, why was that? - Would you have preferred to do this task differently? How? What is advantage of your suggested approach? - If we do a larger study with more schools, should we keep or change the way we ask schools to do this task?   - How else could we do it? |
| --- |

And now our final questions.

| **Final questions**   - Overall, how would you describe your school’s experience of taking part in this study? Why do you say that? - If we do a larger study (with more schools) would you or a representative from your school like to be a member of our advisory board? - Apart from the suggestions you have already made, how can we encourage schools to take part in the future? - Are there any schools in your area that you think we should include a larger study? - Is there anything else you want to say? |
| --- |
