# Supplementary material for: Conducting tobacco control surveys among schoolchildren in Bangladesh, India and Pakistan: A feasibility study
Source: PLOS Glob Public Health. 2024 Oct 3;4(10):e0003784. doi: 10.1371/journal.pgph.0003784 (PMC11449278; doi:10.1371/journal.pgph.0003784)
Supplement: Attachment — Submitted filename: Response to Reviewers.docx [file pgph.0003784.s008.docx]

**'Response to Reviewers'**

We would like to thank the peer reviewers for their valuable comments. Based on their comments, we made necessary changes to our manuscript. The responses to each comment are here.

| Reviewers' comments: | Reviewer's Responses to Questions |
| --- | --- |
| Reviewer #1: Thank you for the opportunity to review this manuscript. It presents an important topic on feasibility of conducting ST surveys in school going youth in 3 Asian countries. This topic is important the data would be useful for interventions. Overall the manuscript is well written.  I suggest that authors highlight the issue of addressing the issue of biased based on sex that was apparent from the findings. | Thanks for pointing out the issue of bias based on sex due to social acceptability of tobacco use by sex in these countries. As this paper is on the feasibility of conducting a survey, we highlighted this issue from that angle.  In the Discussion we added, ‘The social acceptability of tobacco use by gender was highlighted in our study as we observed that the headteacher of a girls' school was reluctant to give permission to take part considering that the girls do not use tobacco and not seeing tobacco as relevant to their students. However, emphasising the relevance of tobacco research to both boys and girls, we encourage all schools, including girls’ schools, to take part. In our study, 61.8% of respondents were boys, and 48.2% were girls.’  In this paper, we are not presenting any data on levels of tobacco use by sex, as this was not the focus of this study. We will present the findings of the survey by country and sex in a separate future paper. |
| Reviewer #2: |  |
| - School selection and recruitment- it’s not clear the criteria that was used to select the administrative areas, subdistricts, and schools. Its mentioned that was purposively, but based on what criteria? Prevalence of tobacco usage, tobacco sold in that region, other? | Thanks for pointing that. We now added the criteria. Now mentioned in the method that ‘A three-stage stratified sampling strategy was used to select eight schools per country. First, we purposively selected two administrative areas from each country. Then from each administrative area, we purposively selected one urban and one rural sub-district. We purposively selected the administrative areas and subdistricts based on the location and data collection facility of our research partner organizations in India, Pakistan (Karachi and Peshawar), and Bangladesh. Finally, in each selected sub-district, we identified schools meeting the inclusion criteria (following mainstream curricula, with year 6-8 classes to allow for follow-up) and stratified them as public or private. Schools were then randomly selected using a number generator in STATA 14.’ |
| - ´One FGD per class was the intention, but due to time constraints, one FGD with students from all three classes was conducted…´ how many students were included in each FGD? | We have now added the information: ‘One FGD per class was the intention, but due to time constraints, one FGD with students from all three classes was conducted in four schools (two in Bangladesh, two in Pakistan, 6-8 children per FGD), and one FGD with one class per school (four schools in India, 8 children per FGD).’ |
| - Who was conducting the interviews and FGDs? Please clarify. What was his/her training? | We have now added the information: ‘One to two members of the research team in each country collected the data. They had mixed levels of qualitative research experience and so were trained and supervised by a senior qualitative researcher (CJ) who held cross-country meetings with researchers and provided training to ensure good data collection practice and consistency across countries, and checked the early transcripts.’ |
| - It would have been interesting to have a group discussion with the parents to understand their perception among the consent process. | We agree with this comment and we add this as a limitation in the discussion section: ‘Second, due to logistical and time constraints, we could not involve parents in the feasibility study. We did not ask parents for their views on the study, their perception of the consent process, and how to improve consent processes, relying instead on school staff and student recommendations. On reflection, this was an oversight, and we would seek to capture their important perspectives in future research.’ |
| - First time mentioning FCTC, please include the abbreviation description. | We now added the abbreviation description of FCTC, when it was first used in the Introduction: ‘These require evidence-based tailored strategies akin to those in the WHO Framework Convention on Tobacco Control (FCTC).’ |
